# Genetics in Ischemic Stroke: Current Perspectives and Future Directions

**DOI:** 10.3390/jcdd10120495

**Published:** 2023-12-13

**Authors:** Ka Zhang, Shaun S. E. Loong, Linus Z. H. Yuen, Narayanaswamy Venketasubramanian, Hui-Lin Chin, Poh San Lai, Benjamin Y. Q. Tan

**Affiliations:** 1 Division of Neurology, Department of Medicine, National University Hospital, Singapore 119074, Singapore; ka_zhang@nuhs.edu.sg; 2 Cardiovascular-Metabolic Translational Research Program, Yong Loo Lin School of Medicine, National University of Singapore, Singapore 119077, Singapore; e0500915@u.nus.edu; 3 Department of Medicine, Yong Loo Lin School of Medicine, National University of Singapore, Singapore 119077, Singapore; 4 Raffles Neuroscience Centre, Raffles Hospital, Singapore 188770, Singapore; ramani_nv@rafflesmedical.com; 5 Khoo Teck Puat National University Children’s Medical Institute, National University Hospital, Singapore 119074, Singapore; paechin@nus.edu.sg; 6 Department of Paediatrics, Yong Loo Lin School of Medicine, National University of Singapore, Singapore 119077, Singapore; paelaips@nus.edu.sg

**Keywords:** genetics, ischemic stroke, stroke recovery, genome-wide association studies (GWASs), pharmacogenetics, pharmacogenomics

## Abstract

Ischemic stroke is a heterogeneous condition influenced by a combination of genetic and environmental factors. Recent advancements have explored genetics in relation to various aspects of ischemic stroke, including the alteration of individual stroke occurrence risk, modulation of treatment response, and effectiveness of post-stroke functional recovery. This article aims to review the recent findings from genetic studies related to various clinical and molecular aspects of ischemic stroke. The potential clinical applications of these genetic insights in stratifying stroke risk, guiding personalized therapy, and identifying new therapeutic targets are discussed herein.

## 1. Introduction

Stroke is the most common cause of disability and is the second leading cause of death, with its worldwide impact on health escalating [1]. Ischemic stroke is a heterogeneous condition influenced by a combination of genetic, environmental, and lifestyle-related risk factors. A family history of stroke is associated with a higher prevalence and incidence of stroke. This has been found to be associated with shared exposures to risk factors such as dietary choices, lifestyle habits, and genetic predisposition [2].

Genetics has been found to play a significant role in the development of stroke, particularly those that manifest at an early age. Previous studies have found that the heritability of ischemic stroke is approximately 37.9%, with levels varying considerably in terms of the specific stroke subtype: 40.3% for large-vessel disease, 32.6% for cardioembolic, and 16.1% for small-vessel disease [3,4]. The current literature has identified numerous genes associated with ischemic stroke through monogenic and polygenic underpinnings. Monogenic etiologies account for 1–5% of all ischemic strokes, while polygenic etiologies are more common, with various risk factors of and ischemic stroke itself having been found to have polygenic associations in large-scale genomic studies [5,6]. However, the precise mechanisms and causal factors underpinning the development of stroke are highly complex and not fully characterized [7].

Genetic factors are known to impact various aspects of ischemic stroke, including the alteration of individual stroke occurrence risk, modulation of treatment response, and the effectiveness of post-stroke functional recovery. This article aims to review the most recent findings from genetic studies related to various clinical and molecular aspects of ischemic stroke.

## 2. Mechanisms of Ischemic Stroke and Stroke Genetics

**Figure 1:**
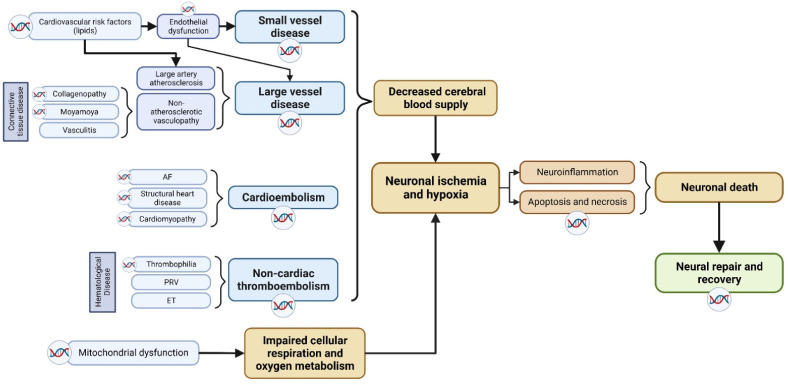
Breakdown of the pathophysiological mechanisms of ischemic stroke. Potential genetic factors based on underlying stroke mechanism are highlighted, including various subtype-based molecular pathways that contributes to neuronal death. The double-helix sign highlights mechanisms which have been identified to have genetic underpinnings. AF: atrial fibrillation; PRV: polycythemia rubra vera; ET: essential thrombocythemia.

As shown in Figure 1, the pathophysiology of ischemic stroke involves two critical stages: (i) the disruption of cerebral blood supply, and (ii), the subsequent development of cerebral tissue hypoxia and necrosis. In the context of ischemic stroke, an initial reduction in cerebral blood supply and/or impaired metabolism of oxygen, glucose, and lipids leads to localized cerebral ischemia. This is followed by a cascade of downstream pathophysiological processes, including excitotoxicity, acidotoxicity, ionic imbalances, oxidative and nitrative stress, neuroinflammation, and apoptosis, among others, all of which ultimately culminate in neuronal death [8]. Following the onset of ischemic stroke, the commencement of a complex neural repair process gradually leads to the functional recovery of patients. Hence, genes involved in either the pathophysiological pathway or the post-stroke neuronal repair pathway may potentially influence the occurrence, severity, and outcome of ischemic stroke.

We delineate the potential genetic factors that could give rise to the described mechanistic phenotypes based on underlying stroke mechanisms.

### 2.1. Cerebral Small Vessel Disease

Cerebral small vessel disease (CSVD) is a vascular disorder affecting cerebral arterioles, veins, and capillaries with diameters ranging from 50 to 400 μm. These vessels supply the cerebral white matter and deep grey matter, with disease typically manifesting as lacunar strokes, vascular parkinsonism, and cognitive impairment [9,10,11]. CSVD contributes to approximately 20% of all strokes, including 25% of ischemic strokes [12,13]. CSVD is related to common cardiovascular risk factors such as hypertension, diabetes mellitus, dyslipidemia, and smoking [14]. In young patients, CSVD due to a hereditary cause is more common, and manifests with leukoencephalopathy changes seen on brain magnetic resonance imaging [15]. Cerebral autosomal-dominant arteriopathy with stroke and ischemic leukoencephalopathy (CADASIL) is the first and most common inherited CSVD syndrome described in the literature. CADASIL arises from mutations in the *NOTCH3* gene located on Chromosome 19p13, and is inherited in an autosomal-dominant manner [16]. Pathogenic variants in *NOTCH3*, particularly variants involving alteration of conserved in cysteine residues, induce nonatherosclerotic, amyloid-negative vascular changes primarily affecting small penetrating arteries, arterioles, and brain capillaries [17,18]. Since the identification of *NOTCH3* as a key modular of CADASIL, more inherited causes of CSVD have been identified, including pathogenic variants in genes relating to cerebral autosomal recessive arteriopathy with subcortical infarcts and leukoencephalopathy (CARASIL) and *COL4A1/A2*-related disorders, amongst others. These genes are responsible for preserving the structural and functional integrity of small-vessel endothelia. Pathogenic mutations in these genes can disrupt small vessel function, ultimately resulting in CSVD [19].

### 2.2. Large Vessel Disease

Large vessel disease (LVD) accounts for 15–20% of all ischemic strokes [20,21]. LVD can be further classified into two major categories: large artery atherosclerosis (LAA) and nonatherosclerotic vasculopathy. Large artery atherosclerosis has traditionally been closely associated with established cardiovascular risk factors, such as diabetes mellitus, hypertension, and dyslipidemia [20]. Consequently, genes linked to cardiovascular risk factors, particularly those involved in lipid metabolism, can heighten the risk of atherosclerosis. Moreover, genes related to endothelial or systemic inflammation can modulate the risk of ischemic stroke by altering the progression of atherosclerosis [22,23]. Genes associated with ischemic stroke are numerous, most commonly for familial hypercholesterolemia with Low-Density Lipoprotein Receptor gene (*LDLR*), Apolipoprotein B gene (*APOB*), *LDLRAP1*, and *PCSK9*. Other lipid metabolism pathway genes implicated include Apolipoprotein E gene (*APOE*), *ATP Binding Cassette Transporter 1* gene (*ABCA1*), and *SCARB1* gene [24]. Associated non-lipid-metabolism-related genes include Transforming Growth Factor Beta 1 Gene (*TGFB1*), Toll-Like Receptors (*TLR*) and Scavenger Receptor (SR) genes, Secreted Phosphoprotein Gene (*SPP1*), *Tumor* Necrosis Factor Receptor Superfamily Member 11b Gene (*TNFRSF11B*), and genes of the Matrix Metalloproteinase (*MMP*) family [24].

In nonatherosclerotic vasculopathy, genes associated with collagenopathy and connective tissue disorders can give rise to structural irregularities in large blood vessels, affecting both the extracellular matrix and smooth muscle contractile components. Vascular dissection, and hemorrhage have been found to be associated with an increased risk of ischemic stroke. Nonatherosclerotic vasculopathy encompasses conditions like Marfan syndrome, fibromuscular dysplasia, and Moyamoya disease. Table 1 lists monogenic disorders in the context of LVD. Many candidate genes have also been reported to carry polygenic risks for LVD including *PCMTD1*, *HDAC9*, *MTHFR*, *TCN2*, *CCER2*, *MRV1*, *PHACTR1*, *CYP11B2*, *PDE4D*, *ADIPOQ*, *LPL*, and *MMP9* [25,26,27,28,29,30].

### 2.3. Cardioembolic Stroke

Cardioembolic stroke (CES) accounts for 20% of all ischemic strokes. Atrial fibrillation (AF) is most common cause of cardioembolic stroke [31]. Genome-wide association studies (GWASs) have identified several genetic loci associated with AF. These loci have been mapped to genes, the function of which can be further categorized into ion channel and non-ion channel. Ion channel genes include potassium channel (*KCNQ1*, *KCNE2*, *KCNE5*, *KCNJ2*, *KCNA5*) and sodium channel genes (*SCN5A*, *SCN1B*, *SCN2B*) [32]. Non-ion channel genes include *NUP155*, *GJA5*, *NPPA* gene [32] and *PITX2*, *ZFHX3*, *ZNF566*, and *PDZK1IP1* [33,34,35]. Nonetheless, not all the genes highlighted above have been shown to be associated with ischemic stroke. Thus far, only four genes (*PITX2*, *ZFHX3*, *ZNF566*, and *PDZK1IP1*) were reported to be associated with both AF and cardioembolic stroke [35].

### 2.4. Embolic Stroke of Undetermined Source

Embolic stroke of undetermined source (ESUS) was a construct introduced in 2014 as a working definition to identify patients with non-lacunar cryptogenic ischemic strokes, in whom embolism was suspected to be the likely stroke mechanism, but with no identifiable cardioembolic source. ESUS accounts for up to 30% of all ischemic strokes [36]. The causes of ESUS are heterogeneous, including non-stenosing carotid plaques, aortic atherosclerotic plaques, pro-thrombotic states, patent foramen ovale with paradoxical thromboemboli, covert occult atrial fibrillation, and left ventricular dysfunction [37]. The precise identification of the underlying cause of ESUS remains challenging. Blanket anticoagulation strategies have resulted in no benefit over an antiplatelet agent for secondary stroke prevention in ESUS patients, owing to the challenges in identifying the etiologies of ESUS in patients [37]. Genomic studies aim to bridge this diagnostic and prevention conundrum. Several genomic studies investigating ESUS cohorts have shown promising advances in knowledge for the precise elucidation of underlying causal mechanisms. In a large-scale GWAS, Marios Georgakis et al. analyzed the genetic architecture of ESUS in 16,851 ischemic stroke cases and 32,473 controls, identifying 19 shared loci of ESUS with LAA, 2 with CES, and 5 with CSVD [38]. In a separate study, Lu-Chen Weng et al. [39] evaluated AF and stroke risk in 26,145 individuals of European descent, utilizing genomic sequencing data. They generated polygenic risk score (PRS) for AF, and subsequently incorporated PRS with clinical risk scores to differentiate cardioembolic stroke from non-cardioembolic stroke. The combined scoring utilizing the AF PRS and clinical risk factors was found to modestly improve the discrimination of cardioembolic from non-cardioembolic strokes, as well as providing an enhanced reclassification of stroke subtypes.

### 2.5. Mitochondrial Dysfunction

Mitochondrial dysfunction impairs tRNA function and mitochondrial protein synthesis, affecting respiratory chain metabolism, eventually leading to multisystem dysfunction, including central nervous system dysfunction. MELAS (mitochondrial encephalopathy, lactic acidosis, and stroke-like episodes) is the most-well-characterized mitochondrial disorder associated with stroke. Affected individuals often present with stroke-like episodes before the age of 40, and can be affected by muscle weakness, hearing loss, and other systemic manifestations. The *MT-TL1:m.3243A>G* variant is the most common pathogenic variant implicated in MELAS [40]. Some common mitochondrial variants implicated in MELAS are shown in Table 2.

### 2.6. Haematological Disorders

Ischemic stroke can be caused by or associated with various hematological diseases due to hypercoagulability, hyperviscosity, and thromboembolism. Various hematological diseases have been shown to have Mendelian inheritance, with disease causing mutations identified in patients. Examples include sickle cell disease (*HBB*), essential thrombocytosis (*JAK2*), polycythemia rubra vera (*JAK2*), and hereditary thrombophilia (*F2*, *F5*).

## 3. Approaches to the Study of Genetics in Ischemic Stroke

There are various approaches that have been employed to examine the genetic underpinnings behind ischemic stroke. Of these, two key approaches are described: (i) linkage analysis and (ii) genome-wide association studies (GWASs).

### 3.1. Linkage Analysis

Linkage analysis is used in genomic analysis to identify regions of the genome that are co-inherited in twins or families with multiple affected members. Genetic variants that co-occur with ischemic stroke serve as genetic markers to characterize the inheritance of genetic material across generations within affected individuals within a family. By comparing the pattern of inheritance of these genetic markers with the pattern of disease transmission within families, researchers calculate linkage scores to estimate the connection between a genetic variant and ischemic stroke [41,42]. Subsequently, the region or locus of interest can be subjected to further sequencing to identify specific genetic causes of the disease. Linkage analysis has been effectively employed in the study of Mendelian disorders like CADASIL and CARASIL [43,44]. While linkage analysis is a robust tool for mapping Mendelian disorders, including monogenic ischemic stroke, it does have certain limitations, especially when studying polygenic disorders. Firstly, it necessitates a large family sample size, making it typically underpowered for polygenic analysis, as well as the identification of genetic variants with low penetrance or effect sizes [45]. Secondly, there may be recall bias in the collection of family history data. Finally, ischemic stroke is a disease with significant genetic and phenotypic heterogeneity, reducing the effectiveness of discovery of linkage analysis. Gene–gene and gene–environment interplay are poorly understood in ischemic stroke, which additionally contributes to the heterogeneity of disease [46]. This heterogeneity can complicate the interpretation of linkage signals.

### 3.2. Genome-Wide Association Studies (GWASs)

GWASs are a powerful and comprehensive approach used in population-level genetic research to identify genetic variants associated with complex diseases, including ischemic stroke. Unlike linkage studies, which are family-based and generally focus on variants obeying Mendelian laws, GWASs involves large cohorts of unrelated individuals aiming to identify genetic variants with effects on disease risk. GWASs can be conducted on both common and rare variant sets and can identify genetic variants with small-to-moderate effect sizes. In the context of ischemic stroke, performing a GWAS involves the identification of genomic loci that are associated with ischemic stroke. By quantifying the differences in the genetics between stroke patients with normal controls, statistically significant candidate loci for increased disease risks are identified. Subsequently, candidate loci and genes undergo further downstream analysis, including phenotype-wide association analysis to identify phenotypic pleiotropy, colocalization to identify biologically significant tissues and mechanisms, candidate gene experimentation, and Mendelian randomization to garner more evidence to establish causality. Of note, polygenic scores can be derived from GWAS summary statistics for the purpose of the genetic prediction of ischemic stroke. There are, however, several limitations associated with genome-wide association studies (GWASs):

- GWASs predominantly focus on identifying common genetic variants, often those with minor allele frequencies exceeding 1–5% [47]. While rare-variant GWASs are also performed to identify associations between higher-impact rare variants with disease risk, studies are generally underpowered for such analysis due to the large sample size and case control ratios required for discovery [48]. Novel statistical models and methods have been developed for more robust rare-variant GWAS analysis to address this [49].
- GWASs can establish associations between genomic loci and ischemic stroke. With large numbers of loci being identified through GWASs, key challenges remain in interpreting the biological significance of associated loci, in spite of the large repertoire of available tools and methods [50]. For example, a significant locus may be in a non-coding region [51] or exist in linkage disequilibrium with the true causal variant that was captured during sequencing [52], resulting in challenges with biological interpretation.
- Ischemic stroke GWASs have mostly been carried out in populations of European descent. This limits the applicability of various genomic findings, such as PRS and genomic underpinnings of ischemic stroke. This key limitation is further coupled with the heterogenous nature of ischemic stroke, in which population-specific differences could contribute to limited replicability across ethnicities.

## 4. Genes Related to Ischemic Stroke Occurrence

### 4.1. Monogenic Ischemic Stroke

Monogenic stroke refers to strokes that are caused by a single pathogenic variant. Monogenic strokes generally follow a Mendelian pattern of transmission through generations. These genetic mutations can increase an individual’s susceptibility to stroke or directly contribute to the development of a stroke. Monogenic strokes account for 1–5% of all strokes [5]. Historically, monogenic disorders related to stroke were studied via case-control methods [41]. Linkage analysis (including family history and twin study) and GWASs [41] are commonly used methods to identify key genes implicated in the pathogenesis of stroke. Table 2 summarizes a list of common monogenic causes of ischemic stroke.

### 4.2. Polygenic Ischemic Stroke

In comparison to monogenic disorders, polygenic stroke is influenced by variants in multiple genes with a low-to-moderate effect size, and does not strictly follow a Mendelian inheritance pattern [53]. Polygenic stroke has been reported to account for 38% of all ischemic stroke cases, although this might be a severe underestimation [6,54]. Ischemic stroke-related gene variants each provide a small contribution to the overall stroke risk in a cumulative manner.

To date, there have been multiple large GWASs performed to identify genes related to polygenic stroke (Table 3). In a meta-analysis study from the MEGASTROKE consortium, Aniket Mishra et al. evaluated over 520,000 subjects largely of European ancestry and discovered 89 independent loci with putative causal genes (such as *SH3PXD2A* and *FURIN*) [55]. In a separate multi-ancestry GWAS meta-analysis, Rainer Malik et al. analyzed 521,612 individuals (67,162 stroke cases and 454,450 controls), and found 32 independent loci, of which 22 were novel loci. In further stroke subtype analyses, the authors identified risks loci *EDNRA* and *LINC01492* for large artery disease and *RGS7* and *NKX2*-5 for cardioembolic stroke [56].

These early large-scale GWASs focused mainly on individuals of European ancestry. The results of these GWASs would likely have limited applicability to other ethnicities. To bridge this gap, there are an increasing number of stroke GWASs being performed that focus on non-European ancestries [51]. Most recently, there was a Chinese GWAS consortia effort, STROMICS, that performed whole-genome sequencing on a large cohort of 10,241 stroke patients from China, identifying 77 risk loci associated with ischemic stroke risks, of which >42% were identified as novel. Notably, disparities in allele frequency were observed between Chinese and other populations for 89 variants associated with stroke risk and 10 variants linked to the response to stroke medications. This analysis provided invaluable insights into the genetic underpinnings of ischemic stroke and its treatment responses [57]. In a separate COMPASS study, Keith Keene et al. investigated >22,000 subjects (3734 cases, 18 317 controls) of African ancestry. The authors identified 1 SNP, *rs55931441* near the *HNF1A* gene as a novel variant associated with any ischemic stroke [58].

### 4.3. Polygenic Risk Score

Polygenic Risk Scores (PRS) are statistical metrics derived from the sum of risk alleles across many genetic loci. PRS can provide insights into an individual’s genetic predisposition to this condition, potentially aiding in earlier diagnosis, prevention, and management [53,59]. In the ASPREE study, Johannes T. Neumann et al. evaluated a PRS calculated using 3,219,276 genetic variants to predict the genetic risk of ischemic stroke for a population of 12,792 healthy older individuals. After 5 years of follow up, the authors reported that the PRS was independently associated with ischemic stroke risk, with hazard ratio of 1.41 per standard deviation of the PRS. In a subgroup analysis, the continuous PRS remained an independent predictor for large vessel and cardioembolic stroke subtypes, but not for small vessel stroke [60]. In another study, Gad Abraham et al. also found a similar predictive value of PRS in ischemic stroke prediction [61]. The authors concluded that the PRS is similarly or more predictive compared to clinical risk scores, taking into account risk factors such as family history, blood pressure, body mass index, and smoking status. Other studies have proposed hybrid scores combining PRS with clinical risk factors, resulting in an increased ischemic stroke predictive value [62,63]. The use of PRS can provide useful information about an individual’s stroke risk at an early time point, even before the clinical manifestation of traditional cerebrovascular risk factors. This may help to guide primary prevention and risk stratification of ischemic stroke. However, PRS is population- and ethnicity-specific. The PRS concluded from a certain population may not be directly translatable to another [64]. All in all, the use of PRS for the stratification of stroke risk in clinical contexts should be used with caution, especially with multifaceted interactions between genetic and environmental risk factors for ischemic stroke.

**Table 3:** GWASs investigating ischemic stroke.

Trial Name	Author and Publication Year	Study Type	Number of Subjects	Ethnicity	Genes Found	Remarks
STROMICS [57]	Yongjun Wang et al., 2023	GWAS of all ischemic stroke, LAS, CES, SVS	10,241 cases, control number was not specified	Chinese	77 loci (>42% novel)	Largest Chinese GWAS
GWAS of Intracranial Artery Stenosis (ICAS) [65]	Shogo Dofuku et al., 2023	GWAS of LAS	757 (408 cases and 349 controls)	Japanese	*rs112735431* in *RNF213*	First GWAS of ICAS
MEGAStroke [55]	Aniket Mishra et al., 2022	Meta-analysis	>520,000 (67,162 cases and 454,450 controls)	Multi-ancestry (largely European, East Asia and African)	89 loci (61 novel) (*SH3PXD2A*, *FURIN GRK5* and *NOS3*)	The largest published GWAS meta-analysis
COMPASS [58]	Keith L. Keene et al., 2020	Meta-analysis of any ischemic stroke	>22,000 (3734 cases and 18,317 controls)	African	1 locus confirmed (*rs55931441* near *HNF1A* gene) 29 other potential variants including those mapped to *SFXN4* and *TMEM108* genes	Consortium of Minority Population Genome-Wide Association Studies of Stroke
SiGN Multi-ancestry GWAS [56]	Rainer Malik et al., 2018	Meta-analysis Of any ischemic stroke, LAS, CES	521,612 (67,162 cases and 454,450 controls)	Multi-ancestry (Largely European, also East Asian, African, South Asian, mixed Asian and Latin American)	32 loci(22 novel) *CASZ1*, *WNT2B*, *KCNK3* for any stroke. *CDK6*, *PDE3A*, *PRPF8*, *ILF3*-*SLC44A2* for any ischemic stroke *EDNRA*, *LINC01492* for LAS *RGS7*, *NKX2*-5 for CES	NINDS Stroke Genetics Network (SiGN), MEGASTROKE Consortium
GWAS on SVO stroke [66]	Tsong-Hai Lee et al., 2017	GWAS of SVS	2073 (342 cases and 1731 controls)	East Asian (Han Chinese)	*rs2594966*, *rs2594973*, *rs4684776* in *ATG7*	
CHARGE [67]	Audrey Y Chu et al., 2016	Meta-analysis of any type of stroke	84,961 (4348 cases and 80,613 controls)	European ancestry	*rs12204590* near *FOXF2* associated with risk of all-stroke	Neurology Working Group of the Cohorts for Heart and Aging Research in Genomic Epidemiology (CHARGE) Consortium
MetaStroke [68]	Traylor M et al., 2012	GWAS of LAS, CES	74,393 (12,389 cases and 62,004 controls)	European ancestry	Cardioembolic stroke near *PITX2* and *ZFHX3* Large-vessel stroke at a *9p21 locus* and *HDAC9*	Goal to validate associations and identify novel genetic associations for ischemic stroke and its subtypes
GWAS for large vessel stroke [69]	Hugh S Markus et al., 2012	GWAS of LAS, CES	12,140 (5859 cases and 6281 controls)	European ancestry	*HDAC9* gene for large vessel stroke *PITX2* and *ZFHX3* for cardioembolic stroke	

### 4.4. SARS-CoV-2 Infection and Ischemic Stroke Genetics

Many studies have reported an association between SARS-CoV-2 infection and acute ischemic stroke [70,71,72,73,74]. All ischemic stroke subtypes have been described in patients with SARS-CoV-2 infection. The postulated mechanisms include inflammation, endothelial cell injury, hypercoagulability, and immune-mediated thrombosis [75,76,77,78]. Most recently, several GWASs have investigated the genetic basis underpinning SARS-CoV-2 infection and ischemic stroke. Parodi et al. reported on the shared genetic background between SARS-CoV-2 infection and large artery stroke [79]. They utilized a PRS for COVID-19 susceptibility and tested its association with stroke subtypes using individual- and summary-level genetic data. They found that the SARS-CoV-2 risk-related *ISLR2* co-expression gene network was significantly associated with the genetic risk of large artery stroke. Similarly, Laila et al. constructed and evaluated a PRS in a SARS-CoV-2 infection cohort and GWASs from the MEGASTROKE, GIGASTROKE, and NINDS Stroke Genetics Network cohorts. Three genomic regions, *PITX2*, *HS6ST1*, and *RFFL*, were found to be associated with large artery atherosclerosis (LAA) and cardioembolic stroke (CES). The authors concluded that ischemic strokes occurring after the onset of COVID-19 infection were genetically similar to LAA and CES subtypes [80].

## 5. Genes Related to Stroke Treatment Response: Pharmacogenetics and Pharmacogenomics

Pharmacogenetics and pharmacogenomics are fields of research that explore the relationship between an individual’s genetic makeup and their response to drugs, namely gene–drug interaction [81,82,83]. Pharmacogenetics focuses on how genetic variations in a single or a set of genes influence an individual’s response to drugs, while pharmacogenomics focuses on the function and interaction of multiple genes in relation to drug metabolism and efficacy. Pharmacogenetics and pharmacogenomics are increasingly being used to maximize efficacy, minimize serious adverse events, and optimize the cost-effectiveness of pharmacological therapies for stroke treatment. In ischemic stroke, commonly used drugs include thrombolytics (alteplase), antiplatelets (aspirin, clopidogrel, and ticagrelor), and anticoagulants (warfarin and direct oral anticoagulants (DOACs)). Key genes related to these drugs are listed in Table 4.

### 5.1. Recombinant Tissue Plasminogen Activator (rtPA):

Recombinant tissue plasminogen activator (rtPA) is widely used for intravenous thrombolysis in patients with acute ischemic stroke. Despite the proven efficacy of rTPA, symptomatic intracranial hemorrhage and the lack of early recanalization have remained significant challenges [84], and are commonly associated with long-term functional disability. In the past decade, there has been significant progress in the understanding of the pharmacogenetics of rTPA. Genes associated with an increased recanalization rate and better outcomes after thrombolysis include *PAI-1*, *TAFI*, *IL1B*, *vWF*, and *ACE*. Genes associated with increased hemorrhagic transformation and worse outcomes include *PAI*-1, *MMP9*, *FXIII* and *FXII*, *A2M*, *ZBTB46*, *ACE*, etc. [82]. Interestingly, Gonz’alez-Conejero et al. [85] found that the homozygous Val/Val genotype of *FXIII* gene was associated with a lower frequency of symptomatic intracranial hemorrhage and mortality rate compared with the Leu allele. In a GWAS meta-analysis of 2034 subjects, Elena Muiño et al. reported new genetic loci associated with parenchymal hematoma hemorrhagic transformation; *Rs79770152* mapped to the *RP11*-*362K2*.2:*RP11*-*767I20*.1 gene and *rs13297983* mapped to *PCSK5* were associated with the occurrence of parenchymal hematoma after thrombolysis [86]. Despite the potential of incorporating rTPA pharmacogenetics in clinical practice, the role of genetic testing to assess response to rTPA is not fully established and has not been included in current guidelines.

### 5.2. Antiplatelet Therapy

#### 5.2.1. Aspirin

Aspirin is one of the most commonly used antiplatelets in the treatment of transient ischemic attack (TIA) and ischemic stroke secondary prevention [87]. The usual recommended dose of aspirin for TIA and secondary stroke prevention ranges from 75 mg to 100 mg/day [88]. Despite compliance with aspirin, stroke recurrence is not uncommonly seen. It is estimated that the prevalence of resistance to aspirin in patients with ischemic stroke or TIA ranges from 5% to 65% [88,89,90].

The mechanism of aspirin resistance is multifactorial, including genetic and environmental interplays. Aspirin exerts its antiplatelet effect by irreversibly inhibiting COX-1, leading to the inhibition of thromboxane A2, which will reduce platelet activation and aggregation. Loci mapped to genes *COX*-1 and *COX*-2 and glycoprotein receptors (*P1A1*/*A2*) were found to be associated with aspirin resistance [91].

#### 5.2.2. Platelet P2Y12 Receptor Antagonist, P2Y12 Inhibitor (Clopidogrel and Ticagrelor)

Clopidogrel is another commonly used antiplatelet in TIA and ischemic stroke. It is estimated that the prevalence of resistance to clopidogrel in patients with ischemic stroke and TIA ranges from 28% to 44% [89,90]. Clopidogrel exerts an antiplatelet effect through the irreversible binding of its active metabolite to the P2Y12 class of adenosine diphosphate (ADP) receptors on platelets [92]. Clopidogrel is a prodrug without a direct antiplatelet effect. It is converted into its active metabolite by the CYP2C19 (cytochrome P450 2C19) enzyme. Carriers of CYP2C19*2 or CYP2C19*3 loss-of-function (LOF) variants have been found to exhibit a poorer response to clopidogrel and a higher risk of vascular events. In addition, carriers of the CYP2C19*17 gain-of-function (GOF) allele have a better response, but a higher bleeding risk [93,94]. In the Clopidogrel in High-Risk Patients with Acute Nondisabling Cerebrovascular Events (CHANCE) randomized trial [95], the authors evaluated the efficacy of dual antiplatelets (aspirin + clopidogrel) versus aspirin alone in 2933 ischemic stroke or TIA patients with 1726 (58.8%) *CYP2C19* LoF carriers. Further subgroup analyses showed that among patients with minor ischemic stroke or TIA, the use of clopidogrel plus aspirin compared with aspirin alone reduced the risk of a new stroke only in the subgroup of patients who were not carriers of the *CYP2C19* loss-of-function alleles [96].

Ticagrelor is a next-generation antiplatelet agent that does not require in vivo bioactivation for P2Y12 receptor inhibition [97]. Thus, it is believed that *CYP2C19* LoF allele carriers may still benefit from Ticagrelor usage. The Clopidogrel With Aspirin in High-Risk Patients With Acute Nondisabling Cerebrovascular Events II (CHANCE-2) trial showed that ticagrelor–aspirin combination therapy reduced the risk of stroke compared with a clopidogrel–aspirin combination among carriers of *CYP2C19* loss-of-function (LOF) alleles after a transient ischemic attack (TIA) or minor ischemic stroke [98]. Stroke recurrence within 90 days was significantly lower in the ticagrelor–aspirin group compared to the clopidogrel–aspirin group (7.6% vs. 6.0%, hazard ratio 0.77, 95% CI 0.64 to 0.94; *p* = 0.008), with similar rates of moderate or severe bleeding (0.3%).

### 5.3. Oral Anticoagulants (Warfarin and Direct-Acting Oral Anticoagulants)

Warfarin is an oral anticoagulant widely used in cardioembolic stroke patients with underlying valvular atrial fibrillation (AF) and left ventricular thrombosis. Warfarin exerts its anticoagulation effect by inhibiting the vitamin K epoxide reductase complex subunit 1 (*VKORC1*) enzyme to inhibit vitamin K metabolism. Studies have shown that rs9923231 (*VKORC1*) carriers exhibit an increased risk of adverse bleeding events, whereas carriers of pathogenic *VKORC1* variants have an increased risk of adverse ischemic events [99,100,101]. Warfarin metabolism is dependent on *CYP2C9*, *CYP4F2*, etc. *CYP2C9* and *CYP4F2* variants are associated with poorer warfarin metabolism, and subsequently an increased risk of bleeding [102,103]. Current studies show that the genetic heritability of warfarin response variability is estimated to be as high as 35% [101]. A better understanding of how genetics contribute to the management of inadequate or excessive anticoagulation may pave the way for improvement in interventions to decrease the risk of stroke events and severe bleeding.

Direct-acting oral anticoagulants (DOACs) exert their anticoagulation effects through the direct and selective inhibition of specific coagulation factors. There are several DOACs available, including Dabigatran, Rivaroxaban, Apixaban, Edoxaban, and Betrixaban. Dabigatran is a factor IIa (thrombin) inhibitor, while the others are factor Xa inhibitors. Compared to warfarin, DOACs do not require the regular monitoring of blood anticoagulation levels and can be administered in fixed doses with fewer drug–drug interactions. Gene variants related to DOACs are listed in Table 4 [104,105].

In the ENGAGE AF-TIMI 48 trial [106], Jessica L Mega et al. compared the efficacy of warfarin versus endoxaban in stroke prevention for patients with AF. A pre-specified subgroup analysis on outcomes based on a genetic analysis of variants in *CYP2C9* and *VKORC1* was performed. Based on genotype, subjects were grouped into normal responder, sensitive responder, and highly sensitive responder groups. During the first 90 days, when compared with warfarin, treatment with endoxaban reduced bleeding rates at a higher rate in sensitive and highly sensitive responders compared to normal responders. This study showed that pharmacogenetics may help identify patients with higher risks of side effects from warfarin, and have an increased benefit from the use of DOACs.

## 6. Genetics of Stroke Recovery

Post-stroke recovery is a complex process influenced by various factors, including the topography and severity of the stroke, acute intervention received, time from stroke onset to reperfusion therapy, patients’ comorbidities, and rehabilitation course. Studies have explored genetics as a factor that may contribute to motor recovery, particularly in terms of motor skill improvements and rehabilitation outcomes (see Table 5) [107,108,109,110].

Marina Mola-Caminal et al. [107] found a locus mapped to the *PATJ* gene that was associated with a worse functional outcome at 3 months after stroke. However, the biological mechanisms behind this are unclear. In the GISCOME (Genetics of Ischaemic Stroke Functional Outcome Network) GWAS, Martin Söderholm et al. [108], found a genetic variant (rs1842681) associated with a better modified Rankin score at 3 months after stroke. This was previously reported to be a trans-expression quantitative trait locus for *PPP1R21*, which encodes a regulatory subunit of protein phosphatase 1 in neuronal tissue. This ubiquitous phosphatase is implicated in brain functions such as brain plasticity. This analysis also revealed a few genetic variants such as *NTN4*, *TEK*, and *PTCH1,* which may influence infarct volume and brain recovery. The research into the genetic underpinnings of stroke outcomes and recovery can provide direction for further functional studies to understand the biological and pathophysiological mechanisms of neuronal death and neural repair in ischemic stroke, paving the way for novel biomarkers and therapeutics in stroke recovery.

## 7. Clinical and Translational Applications of Genetics in Ischemic Stroke

The application of genetics in ischemic stroke is a rapidly developing area. Substantial progress in understanding the genetic underpinnings of stroke has laid the groundwork for personalized molecular approaches. Traditionally, stroke was thought to be a heterogeneous condition mainly associated with traditional clinical cardiovascular risk factors. Genetic factors were seen as non-essential and non-modifiable risk factors with very limited influence on stroke management. However, with recent advances, genetics has been shown to play an important role in stroke management and research, including elucidating the underlying mechanism, accurate subtyping, tailoring treatment strategies, predicting risks and outcomes, and identifying novel therapeutic targets through drug discovery and Mendelian randomization approaches [111].

The current applications of genetics in ischemic stroke include the following:

1. Molecular diagnosis, prognosis, and counselling of patients with typical clinical or radiological phenotypes suggestive of a monogenic disorder, e.g., anterior temporal lobe white matter hyperintensities seen in CADASIL.
2. Molecular diagnosis of monogenic disorders in early-onset stroke, stroke with systemic manifestations, or patients with a significant family history.
3. Tailoring antiplatelet therapy using pharmacogenetics approaches, e.g., *CYP2C19* genotyping for clopidogrel.
4. Risk prediction based on common genetic variants using polygenic risk scores.

Currently, there are no available molecular treatments for ischemic stroke. However, pharmacogenomics has started to provide guidance influencing personalized stroke treatment. The genetics of molecular mechanisms of ischemic stroke may provide insight into potential targets for novel drug development and drug re-purposing. The genetics of stroke recovery may help identify genetic mechanisms related to neural repair, which may in turn lead to the discovery of therapeutics that can hasten neural repair.

## 8. Future Directions

**Figure 2:**
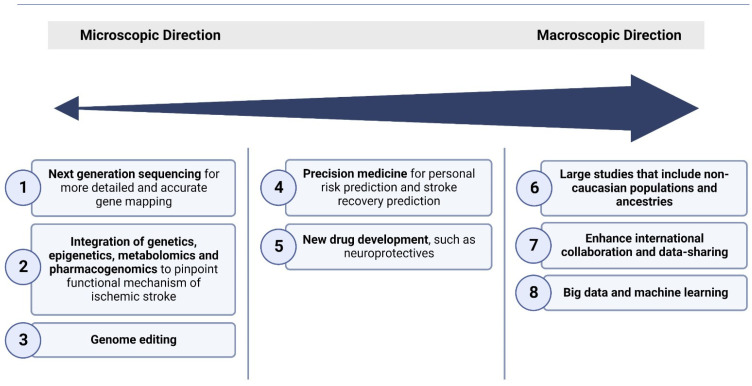
Broad overview of the various approaches used for the elucidation of the molecular underpinnings of ischemic stroke, focusing on current and future directions of genetics approaches.

The future of genetics in ischemic stroke holds great promise, with ongoing research poised to revolutionize our understanding of this complex neurological condition. As science advances and technology evolves, the landscape of genetics in ischemic stroke is rapidly shifting towards exciting and innovative directions that have the potential to transform diagnosis, treatment, and prevention strategies. Figure 2 summarises these directions.

These future directions encompass several key areas:

(1) Next-generation sequencing on a large scale

Genetic studies, including GWASs, are likely to uncover additional genetic variants associated with ischemic stroke risk. These discoveries can provide insights into previously unknown biological pathways and mechanisms underlying the development of the disease, laying the groundwork for further validation and therapeutic angles. There are multiple international efforts that will coalesce to enhance our understanding of common disease genetics, including ongoing work in the UK [112], Estonian [113], Finnish [114], and Japanese [115] Biobanks.

(2) Integration of multi-omics data to understand stroke pathophysiology

Ischemic stroke occurrence and recovery are complex, heterogenous pathophysiological processes influenced by a myriad of genetic, epigenetic, transcriptomic, proteomic, metabolomic, and pharmacogenomic factors. Multi-omics approaches, by integrating multi-omics data, will enable us to have a holistic understanding of the intricate interplay of biological pathways and regulatory networks that drive ischemic stroke onset, progression, and recovery [116]. More research is required in the future to complete the big picture of stroke multi-omics.

(3) Gene therapy

There is no gene therapy available in clinical use for ischemic stroke. However, there are various pre-clinical approaches showing the promising results of gene therapy in acute ischemic strokes [117,118]. Emerging technologies like CRISPR-Cas9 and base editing hold the potential to correct genetic mutations associated with ischemic stroke, potentially preventing the development of the disease in at-risk individuals. However, ethical issues still remain in gene therapy [119].

(4) Precision medicine

With the increase in the availability of an individual’s genetic information, physicians can assess an individual’s genetic predisposition to ischemic stroke and its risk factors, and predict response to stroke treatment and outcome. Clinicians can thus implement personalized prevention and targeted interventions, reduce side effects of medication, and optimize outcomes [120].

(5) New drug development

Genetic research has paved the way for novel therapeutic biomarkers and targets for ischemic stroke. Targeted therapies aimed at the underpinning molecular mechanisms may offer new avenues for treatment, including neuroprotective agents and interventions to reduce stroke burden [118,121,122].

(6) Expanding genetic studies to underrepresented populations

The vast majority of genetic research on the mechanisms underpinning ischemic stroke has primarily focused on European-ancestry populations, leading to an unequal representation of ancestral backgrounds in genetic studies compared to real-world diversity. Recently, there have been emerging initiatives aimed at investigating stroke genetics within African and Asian cohorts [57,58,123,124]. These endeavors, in addition to forthcoming cohorts, hold the potential to substantially enhance our understanding of stroke genetics, helping to bridge the gap in comprehending the underlying mechanisms, discovering biomarkers, and developing therapeutic strategies.

(7) Enhancement of global collaboration

International collaborations and data-sharing initiatives will foster a deeper understanding of the genetic underpinnings of ischemic stroke, particularly in diverse populations, improving the generalizability of research findings. Currently, there are several international stroke genetic consortia, including the International Stroke Genetics Consortium (ISGC) [125], MEGASTROKE Consortium [126], and GISCOME consortium [127].

## 9. Conclusions

In this review, we delve into the current and future role of genetics in understanding ischemic stroke. As our understanding of stroke genetics continues to deepen, there is a growing prospect that ischemic stroke, which was once an unpredictable and devastating disease, can now be envisaged as a condition that is preventable, predictable, and amenable to treatment through various current and future advances in precision medicine. In the meantime, more work is warranted towards uncovering various pathophysiological mechanisms behind the subtypes of ischemic stroke, and towards advancing new biomarkers and therapeutic approaches through evidence-based molecular and clinical research.

## Figures and Tables

**Table 1:** Monogenic causes of ischemic stroke.

Mechanism of Ischemic Stroke	Disease	Related Genes	Inheritance Pattern	Key Clinical Features
Small vessel disease	CADASIL	*NOTCH3*	AD	Migraine, recurrent transient ischemic attacks and lacunar infarcts, vascular cognitive impairment, anterior temporal lobe white matter hyperintensities
	CARASIL	*HTRA1*	AR	Similar to CADASIL, but of autosomal recessive inheritance pattern
	Cathepsin A-related arteriopathy with strokes and leukoencephalopathy (CARASAL)	*CTSA*	AD	Migraine, transient ischemic attack, recurrent strokes, vertigo, dysphagia, cognitive impairment, REM-sleep behavioral disorder
	Retinal vasculopathy with cerebral leukoencephalopathy and systemic manifestations (RVCL-S)	*TREX1*	AD	Visual loss, vascular retinopathy, migraine, cognitive impairment microvascular renal disease, Raynaud phenomenon
	Fabry disease	*GAL*	X-linked recessive	Transient ischemic attack, stroke. May also be associated with other stroke phenotypes including large vessel disease Acroparasthesias, corneal and lenticular opacities, cardiomyopathy
	FOXC1-deletion related cerebral small vessel disease	*FOXC1*	AD	Hearing impairment, cerebellar malformations, white matter hyperintensities on MRI Brain
	Type IV collagenopathy	*COL4A1* and *A2*	AD	Recurrent strokes, seizures, migraine, visual loss, nephropathy, myopathy, arrhythmias, intracranial aneurysms or dolichoectasia
	Pontine autosomal-dominant microangiopathy with leukoencephalopathy (PADMAL)	*COL4A1*	AD	Recurrent lacunar infarcts with predilection to the pons, progressive cognitive impairment
Large vessel disease	Ehlers–Danlos Syndrome Type IV	*COL3A1*	AD	Facial acrogeria, skin fragility and bruising, large and medium vessel artery dissections, including extracranial and intracranial vertebral and carotid arteries
	Pseudoxanthoma elasticum	*ABCC6*	AR	Increased skin elasticity, skin discoloration, ocular angioid streaks
	Marfan syndrome	*FBN1*	AD	Marfanoid features, ascending aorta dissection
	Moyamoya disease	*RNF213*	AD or AR	Steno-occlusive disease of the terminal internal carotid artery with “puff of smoke” collaterals
Haematological disorders	Sickle cell disease	*HBB*	AR	Pain crises, seizures, myelopathy, anemia, thrombosis Can be associated with both large vessel and small vessel infarcts
	Polycythemia rubra vera (PV)	*JAK2*	AD or AR	Ischemic stroke due to hyperviscosity state
	Essential thrombocythemia (ET)	*JAK2* *MPL*	AD or AR	Ischemic stroke due to hyperviscosity state
	Homocystinuria Hyperhomocysteinemia	*CBS* *italic>MTHFR*	AR	Raised plasma homocysteine level, atherosclerosis, thrombosis Classic homocystinuria: ectopia lentis, tall stature, pectus excavatum, developmental delay, thromboembolism
Hereditary thrombophilias	Factor V Leiden mutation (FVL)	*F5*	AD	Can be associated with small vessel, large vessel, and embolic infarcts
	Prothrombin gene mutation (Factor II mutation)	*F2*	AD	
	Protein C deficiency	*PROC*	AD or AR	
	Protein S deficiency	*PROS1*	AD or AR	
	Antithrombin III deficiency	*SERPINC1*	AD	
	Paroxysmal nocturnal hemoglobinuria (PNH)	*PIGA*	X linked	Complement-mediated hemolysis. Can be associated with small vessel, large vessel and embolic infarcts

**Table 2:** Some common mitochondrial variants related to MELAS.

Variant	Gene	Remarks
*m.3243A>G m.3271T>C m.3252A>G*	*MT-TL1*	Encodes the transfer RNA for leucine. This mutation results in the disruption of the function of mitochondrial tRNA, affecting protein synthesis within the mitochondria.
*m.13513G>A*	*MT-ND5*	A part of complex I of the mitochondrial respiratory chain.
*m.8344A>G*	*MT-TK*	Encodes the transfer RNA for lysine. This mutation is more commonly associated with another mitochondrial disorder known as MERRF (Myoclonic Epilepsy with Ragged Red Fibers), but it can sometimes cause MELAS or a MELAS/MERRF overlap syndrome.
*m.14453G>A*	*MT-ND6*	Associated with MELAS phenotype with dystonia.
*m.13042G>A*	*MT-ND5*	Associated with MELAS syndrome with a cardiomyopathy phenotype.
*m.8993T>G/C*	*MT-ATP6*	Associated with NARP (Neuropathy, Ataxia, and Retinitis Pigmentosa) syndrome, but can sometimes produce MELAS symptoms.

**Table 4:** Pharmacogenes related to medications used in ischemic stroke treatment.

Drug		Related Genes
Recombinant tissue plasminogen activator (rtPA) or Alteplase		Increased recanalization: *PAI*-1, *TAFI*, *IL1B*, *vWF*, *ACE* Increased hemorrhagic conversion: *PAI-1*, *MMP9*, *FXIII* and *FXII*, *A2M*, *ZBTB46*, *ACE*
Aspirin		*COX-1*, *COX-2*, *P1A1/A2COL1A1*, COL1A2, vWF, ITGA2B, UGTIA6*2, *ADRA2A*, *TXBA2R*, *PLA2G7*
Clopidogrel		Hepatic metabolism: *CYP3A4*, *CYP1A2*, *CYP2C19* Intestinal absorption: *ABCB1* Glycoprotein: *ABCB1* Platelet surface receptors: *P2Y1*, *P2Y12*
Ticagrelor		*SLCO1B1*, *UGT2B7*, *CYP3A4*
Warfarin		*VKORC1*, and *CYP2C9*
DOAC	Dabigatran	Activation: *CES1*, *CES2* Transport: *ABCB1* Metabolism: *UGT1A9*, *UGT2B7*, *UGT2B15*
	Rivaroxaban	Transport: *ABCB1*, *ABCG2* Metabolism: *CYP3A4*/5,*CYP2J2*
	Apixaban	Transport: *ABCB1*, *ABCG2* Metabolism: *CYP3A4*/5,*CYP2J2*,*CYP1A2*
	Edoxaban	Transport: *ABCB1*, *SLCO1B1* Metabolism: *CES1*, *CYP3A4*/5
	Betrixaban	Transport: *ABCB1* Metabolism: CYP450-*independent* hydrolysis

**Table 5:** Studies of stroke recovery genetics.

Trial Name	Author and Publication Year	Study Type	Number of Subjects	Ethnicity	Genes Found
Post Stroke Motor Recovery GWAS: A Domain-Specific Approach [110]	Chad M Aldridge et al., 2023	GWAS	2100 cases	Not mentioned	No genome-wide significant loci found. 115 SNPs’ subthreshold associations were identified. *CLDN23* gene had the most convincing association, which affects blood–brain barrier integrity, neurodevelopment, and immune cell transmigration
Six GWAS-linked hot loci on stroke outcome [109]	Ruixia Zhu et al., 2021	GWAS	982 cases	Northern Chinese	*ALDH2 rs10744777*, *HDAC9 rs2107595*, ABO *rs532436* (associated with increased stroke recurrence), *PATJ rs76221407*, *LOC105372028 rs1842681*, *PTCH1 rs2236406* (associated with poor stroke outcome)
GWAS of functional outcome (GISCOME) [108]	Martin Söderholm et al., 2019	Meta-analysis	6165 cases	Europe, US, and Australia	*LOC105372028 rs1842681* was associated with brain plasticity and good stroke outcome
PATJ Variants Are Associated with Worse Ischemic Stroke Functional Outcome [107]	Marina Mola-Caminal et al., 2019	Meta-analysis	>5000 cases	European ancestry	*PATJ* (Pals1-associated tight junction) gene (associated with worse functional outcome at 3 months after stroke)

## Data Availability

Not applicable.
